# Supplementary material for: Melanoma Brain Metastases Patient-Derived Organoids: An In Vitro Platform for Drug Screening
Source: Pharmaceutics. 2024 Aug 5;16(8):1042. doi: 10.3390/pharmaceutics16081042 (PMC11360789; doi:10.3390/pharmaceutics16081042)
Supplement: Supplementary file 1 [file pharmaceutics-16-01042-s001.zip › pharmaceutics-3115502-supplementary.pdf]

Supplmentary Table S1: MBM-PDOs culture media.

Reagent name	Company	Final concentration
L-WRN cells' conditioned media	ATCC, Virginia, USA	1:1 ratio
Advanced DMEM/F12	Gibco, fisher scientific, Grand Island, USA	1x
penicillin/streptomycin (100x)	Carl Roth, Karlsruhe, Germany	2%
GlutaMax (100x)	Carl Roth, Karlsruhe, Germany	2x
HEPES solution	Carl Roth, Karlsruhe, Germany	20mM
B27 supplement (50x) without Vitamin A	Merck, Darmstadt, Germany	2x
N2 supplement	Merck, Darmstadt, Germany	2x
[Leu15]-Gastrin I human	Merck, Darmstadt, Germany	20 nM
N-Acetyl-L-cysteine	Acros Organics, Geel, Belgium, Thermo Fisher Scientific	2.5 mM
Nicotinamide	Sigma-Aldrich, St. Louis MI, USA	20 mM
Recombinant human EGF	Peprotech, Rocky Hill, NJ, USA	50 ng/ml
SB202190	Peprotech, Rocky Hill, NJ, USA	10 µM
A8301	Sigma-Aldrich, St. Louis MI, USA	0,5 µM
Y27632 dihydrochloride	Biogems, Westlake Village, CA, USA	10 µM
Normocin	InvivoGen, San Diego CA, USA	100 µg/mL

Supplmentary Table S2: Quantification of Ki67 staining.

**Case**	**% of Ki67 positive nuclei**	
	**Primary tumor tissue**	**Organoid tissue**
**SA1**	70%	60%
**SA3**	50%	50%
**SA12**	40%	90%
**SA17**	10%	10%
**SA20**	5%	5%
**SA34**	50%	45%
**SA41**	60%	50%
